# Can Nuclear Power Products Mitigate Greenhouse Gas Emissions? Evidence from Global Trade Network

**DOI:** 10.3390/ijerph19137808

**Published:** 2022-06-25

**Authors:** Tingzhu Li, Debin Du, Xueli Wang, Xionghe Qin

**Affiliations:** 1 Institute for Global Innovation & Development, East China Normal University, Shanghai 200062, China; litingzhuz@foxmail.com; 2 School of Urban and Regional Science, East China Normal University, Shanghai 200062, China; 3 Institute of Central China Development, Wuhan University, Wuhan 430072, China; 4 Department of Geography, National University of Singapore, Singapore 117570, Singapore; 5 School of Economics, Hefei University of Technology, Hefei 230009, China; xhqin@re.ecnu.edu.cn

**Keywords:** nuclear power products, greenhouse gas emissions, international trade, network position

## Abstract

Since its birth, nuclear power has been a hot topic of academic research while being subject to much controversy. As a new green energy source with zero greenhouse gas (GHG) emissions, nuclear power plays a vital role in combatting global climate change. Based on global databases and various empirical analysis methods, this study aimed to explore the changes in the global nuclear power product trade (GNT) network and its impact on GHG emissions from 2001 to 2018. The main findings are summarized as follows. (1) Global trade in nuclear power products and GHG emissions showed a non-linear and fluctuating growth during the research period. The geographical pattern of GNT not only has prominent spatial heterogeneity, but it also has some spatial reverse coupled with the spatial distribution of global GHG emissions. (2) The overall regression analysis finds that nuclear power product trade had a significant suppressive effect on global GHG emissions and had the greatest influence among all the selected variables. (3) As for the impact of the GNT network on GHG emissions, nuclear power product trade was better able to curb GHG emissions in countries with the dominate positions compared to those with affiliated positions, which reflects the heterogeneous effect of nuclear power product trade on GHG emissions. These results provide further evidence for the dialectical debate on whether nuclear power products contribute to GHG emissions reductions. This paper also provides corresponding recommendations for policymakers.

## 1. Introduction

Global warming has long aroused people’s widespread concern. The international response to this issue began with the adoption of the United Nations Framework Convention on Climate Change (UNFCCC) in 1992. The UNFCCC’s principal framework for action aimed to stabilize atmospheric concentrations of GHGs to avoid “dangerous anthropogenic interference” with the climate system [1]. With the entry into force of the Kyoto Protocol to the UNFCCC in February 2005, the efforts of the international community have reached a climax. The protocol requires developed countries and economies in transition to reduce their combined emissions of GHG by an average of 5.2% from 1990 levels between 2008 and 2012 (the first commitment period) [2]. Therefore, it has become a crucial issue of human survival to explore how to slow down greenhouse gas emissions to curb the process of global warming. 

During the last decades, energy exploitation contributed to socioeconomic development and changed people’s lives radically. However, the use of traditional energy sources has a negative impact on the environment. For instance, while fossil fuels supply more than 68% of the commercial electricity, the huge dangers of climate change are caused mainly by fossil fuel combustion [3]. In this context, renewables, which are considered as effectively carbon-free, have sparked interest worldwide. Increased renewable energy utilization in 2018 exerted an even more significant influence on GHG emissions, preventing 215 million tons of emissions [4]. A large share of renewable energy in the energy portfolio will not only encourage economic growth and reduce GHG emissions, but it will also help to accomplish the Sustainable Energy goal, as well as increase energy efficiency and decrease energy dependence [5]. Although renewable energy is a significant part of energy policy to increase/decrease efficiency/dependence, still, in many countries, no significant improvement has been observed in achieving the goals of environmental sustainability and the growth of renewable energy. Because there are certain problems with the deployment of renewable energy, such as lack of technology and infrastructure, high investment costs and inadequate political and public awareness and adaptation choices for climate change remain significant challenges [6,7,8]. Therefore, with the deepening of people’s understanding of environmental problems and the further deterioration of energy problems, the increasing urgency of mitigating impending global warming has sparked renewed interest in clean energy options. Among them, nuclear power is receiving a massive consideration from policymakers [9] because it takes part not only in mitigating carbon emissions, but it also plays an essential role in economic growth as well with lower costs of electricity generation [10,11]. There is no doubt that the shift toward clean energy is highly important to fight global warming and stabilize climatic conditions. Nuclear energy can deal with high energy prices and decrease dependence on energy imports, as well as decrease GHG emissions [12]. Switching from fossil fuels to a larger share of nuclear energy reduces pollution and would decrease considerably the impacts of global climate change on the quality of life of the residents [13]. This all suggests that nuclear energy seems to be one of the best alternatives. In the current situation, there are still some disputes about nuclear power itself, but the fact is that nuclear power hardly emits GHG in practical applications, and it is one of the electricity-generating options with the lowest emissions in terms of life cycle GHG releases [14]. Indeed, international studies have shown that nuclear power products play crucial roles in combating global warming [15,16]. It is necessary to point out that nuclear power has been excluded from the Kyoto Protocol’s mechanisms as a qualified technology [2].

Further, since the world is becoming “spiky” [17,18], there are significant regional differences in the spatial distribution of nuclear power products. This kind of spatial inequality is bound to have varying degrees of impact on the governance of global warming. We must first learn how to assess changes in the regional heterogeneity of nuclear power products before we can begin our investigation. International trade is the most intuitive embodiment of the spatial flowing of products all over the world, so we chose the data of GNT to depict the world layout of nuclear power products. Therefore, it is of reference value to strengthen the research on the spatial-temporal evolution of the GNT and deeply explore its influencing mechanism on GHG emissions, so as to curb global warming and improve the pertinence and effectiveness of governance.

Based on the above background, the research on the impact of the use of nuclear power products on GHG emissions has rapidly become a hot topic in academic circles. However, few scholars conduct systematic research on the global scale, and it is relatively scarce to research the influencing factors of GHG emissions in countries with different positions in the GNT, so this paper intended to conduct in-depth studies on the following core issues: (1) In terms of spatial-temporal characteristics, is there a correlation between GNT and GHG emissions? For instance, are the regions where countries with high trade volumes are clustered low value areas for GHG emissions? (2) What is the impact of the GNT on global GHG emissions in terms of statistical analysis? (3) Does the country’s network position have an impact on GHG emissions? For example, are GHG emissions more restrained by the GNT in the countries with dominant positions? 

The contributions of this study are as follows. First, we used spatial analysis to find a certain spatial inverse coupling phenomenon between GNT and GHG emissions. Second, we investigated whether GNT has a statistically significant inhibitory effect on GHG emissions. Finally, we applied the previous influence mechanism analysis to the countries in different network positions, which not only further confirmed that network position could effectively affect GHG emissions, but also found the heterogeneous characteristics of the effect of nuclear power product trade on GHG emissions. The above findings fill the gaps in existing research and broaden the path for future studies.

The rest of this paper is arranged as follows. We begin with a literature review of the studies of the effects of nuclear power and international trade on GHG emissions in Section 2. Section 3 introduces data and methodology. Section 4 explores the mechanism of the impact of nuclear power product trade on GHG emissions from multiple perspectives. Finally, the conclusions are presented in Section 5.

## 2. Literature Review

The generation of nuclear power dates back to 1942, and Fermi successfully invented the first self-sustaining nuclear reaction at the University of Chicago [19]. Since then, nuclear power technology has developed rapidly. In 2018, 440 nuclear plants supplied 11% of the world’s power, constituting 390 GW of installed capacity generating 2709 TWH of electricity [20]. In the US alone, which has 29.2% of the world’s reactors, nuclear facilities accounts for 19% of the national electricity generation. In France, 79% of electricity comes from nuclear sources, and nuclear energy contributes to more than 20% of the national power production in Germany, Japan, South Korea, Sweden, Ukraine, and the United Kingdom [21]. Before formally starting the literature review, a specific overview of the use of nuclear power in different countries is necessary. In developed countries, nuclear power seems to be gradually being resisted. The Three Mile Island nuclear accident in 1979 and the Chernobyl accident in 1986 led to an anti-nuclear movement in several countries around the world, and Germany was one of the countries most affected by the anti-nuclear movement, which led to no new commercial reactors being built in Germany after 1989. After the Fukushima nuclear meltdown in Japan in 2011, the German government announced that it was suspending plans to extend nuclear power plants until 2036, influenced by popular will and a reconfiguration of the energy consumption mix, and four months later they announced the closure of eight of its seventeen nuclear reactors, with the remaining nine to be shut down by 2022 [22]. Similarly, France’s nuclear power plants are being gradually closed, but because its nuclear energy share is so high, it is not as aggressive as Germany, but has adopted a policy of gradually scaling back nuclear energy. In 2017, France introduced the Energy Transition Act, which plans to reduce the share of nuclear power from 75% to 50% by 2025 [23]. These actions seem to represent the attitude of developed country governments toward nuclear power. In developing countries, however, the use of nuclear power is quite different. In order to cope with domestic environmental degradation and reduce energy import dependence, the Indian government is vigorously promoting the construction of nuclear power plants and expects to increase the share of domestic nuclear power generation to 25% by 2050, a move that not only relieves domestic electricity demand, but also stimulates energy consumption [24]. China is also a major country in nuclear power applications. Starting with the 11th Five-Year Plan, the Chinese government has made clear its goal of actively promoting the construction of nuclear power. Although the development of China’s nuclear power industry has stalled due to the Fukushima incident, the industry continues to thrive as China’s nuclear power technology is upgraded and domestic market demand increases [25].

### 2.1. The Impact of Nuclear Power on GHG Emissions

Concerning studies on nuclear power and GHG emissions, lots of scholars have made great contributions to the relationship between both of them. The main conclusions are divided into two opposing groups: nuclear power is cleaner/dirtier so that it can/cannot effectively alleviate the pressure of GHG emissions. Therefore, the literature closely related to the issue can mainly be divided into the following two branches.

The first branch is that nuclear power plays a positive role in curbing GHG emissions. Firstly, national governments have recognized the unique ability of nuclear energy to provide solutions to environmental problems [26]. The National Energy Policy Act of 2005 established and authorized the Department of Energy’s (DOE’s) Nuclear Power 2010 Program to stimulate the construction of new nuclear power plants in the US [27]. Social groups and business advocates have listed nuclear power as an important part of solutions aimed at reducing GHG emissions. For example, Patrick Moore, co-founder of Greenpeace, has publicly argued that nuclear power is the only non-GHG emitting energy source that can effectively satisfy global demand [28]. Areva, the world’s only one-stop nuclear shop, claims that one gigawatt of a coal-fired power station emits 6 million tons of CO_2_ a year, while a nuclear power station is quite CO_2_-free [21,29]. Secondly, many researchers have conducted in-depth discussions on this issue by taking some countries as cases. In scientific terms, nuclear energy is not a renewable energy source, but some researchers have made their findings more representative by unifying nuclear energy with renewable energy sources in order to better compare them with non-renewable energy sources. For example, DeLlano-Paz et al. focused on renewable energy power generation in the EU in 2020 and 2030 and suggested that nuclear power is an effective way to meet the government’s emission reduction targets [30]. Sims et al. evaluated the amount of GHG emissions per kWh that electricity generates and concluded that the life-cycle GHG emissions per unit of electricity from nuclear power plants are at least two orders of magnitude lower than those from fossil-fueled electricity generation and comparable to most renewables at near zero in. Hence, they point out that nuclear power generation is an effective GHG mitigation option, especially by way of investments, to extend the lifetime of existing plants [31]. By comparing different kinds of power plants, Abbaspour et al. found that nuclear power is not directly increasing GHG emissions and that nuclear power plants could be a suitable replacement for thermal power plants [1]. Ren et al. established models to conduct a life-cycle analysis of primary-energy consumption and GHG emissions of hydrogen supply chains for fuel-cell vehicles in China [32]. They found that hydrogen production from nuclear power has slightly lower GHG emissions than fossil fuels and that it has the potential to replace fossil energy and thus reduce emissions. 

However, there are certain risks in the use of nuclear power. The most recent dangerous event related to nuclear power is the nuclear leak at Japan’s Fukushima Daiichi nuclear power plant caused by the earthquake and tsunami in 2011. Fukushima uncovered dormant fears among the public about nuclear power and national debates about whether or not nuclear power is an ethical source of energy [33]. Nuclear power generation declined significantly following Fukushima, both in net terawatt hours and share of electricity production [34]. Subsequently, Markandya and Wilkinson calculated that nuclear energy is nearly 54 times safer than gas and claimed that nuclear power should be the preferred power source if safety is a number one concern [35]. Actually, nuclear is safer than other sources; nuclear power needs to be retained and expanded in order to avoid or minimize the devastating impacts of climate change by fossil fuel burning [36,37]. Although there are some hidden dangers in the use of nuclear power, many studies believe that, with future development, nuclear power is going to be a mainstay utility with its pragmatic value [38,39,40]. Therefore, the dissenting minority view should take a back seat to progress—safe, reliable, clean, long-term progress [33].

The second branch is that nuclear power does not effectively curb GHG emissions and has a negative impact on the environment. In the current debate on the impact of nuclear power on GHG, the results of different studies available in the open literature contradict each other, as well as the conclusions drawn from them [41]. That is because many researchers think that the operation of so-called ‘emission-free’ power plants such as nuclear power plants will not lead to global warming, but indirect GHG emissions result from the extraction and transformation of raw materials, the construction of nuclear power plants, and other process steps in the whole life cycle [21,41]. In the Storm van Leeuwen and Smith study, the authors calculated GHG intensities for the present nuclear life cycle, which are reasonably higher than other studies in the literature, which made the authors conclude that it is hardly possible for nuclear power to deliver a significant contribution to the world energy demand in the long term [42,43]. Moreover, other opponents of nuclear power argue that nuclear plants are poor substitutes to other less greenhouse gas-intensive generators [44]. The Oxford Research Group predicts that if the percentage of the world’s nuclear power capacity remains what it is today, nuclear power would produce as much CO_2_ per kWh as comparable gas-fired power stations by 2050, as the grade of available uranium ore decreases [21,45,46]. Maennel and Kim also found that nuclear power has begun phasing out, and the proportion of other renewable and clean energy has been expanded in the energy mix to over 50% since 2011 in Germany [47]. In addition to nuclear power not being able to curb GHG emissions, people are still worried about the environmental harm caused by the leakage of nuclear power plants. Since the Fukushima nuclear disaster in 2011, the use of nuclear power has become extremely controversial, mainly because it may have a disastrous impact on the environment in the event of an accident. Furthermore, permanent disposal of nuclear waste remains a very troublesome issue due to its high cost and the inability to adequately analyze the environmental long-term repercussions [48,49].

### 2.2. The Impact of International Trade on GHG Emissions

In addition to the above research topics, the impact of international trade on GHG emissions has also attracted a “boom” in research. International trade has an impact on global carbon emissions and even global climate through production and consumption activities on a global scale, which in turn affects global GHG emissions. There are two main ways in which international trade affects global GHG emissions. On the one hand, scale, technology, and structural effects of international trade have an impact on GHG emissions. More specifically, the theoretical structure of trade scale effects, technology effects, and structural effects is also known as “Austrian Capital Theory” [50,51]. Werner et al. analyzed SO_2_ concentrations in 43 countries from 1971 to 1996 and found that scale, technology, and structure affect carbon emission levels, suggesting that the degree of openness of international commodity markets affects pollution levels [50]. Managi verified the relationship between trade openness and CO_2_ emissions using panel data for 63 countries from 1960 to 1999, and the results proved that trade liberalization increased CO_2_ emissions [51]. It is unfair to quantify a country’s GHG emissions according to the traditional trade connotation carbon in the process of trade import and export, and more scholars have started to pay attention to the fairness of GHG emission responsibility determination [52,53,54,55]. On the other hand, international trade shifts industries globally and affects global GHG emission patterns [56,57]. International trade, because of its cross-regional and multi-scale trade interactions, should also be considered when considering its impact on GHG emissions and other related climate issues due to industrial shifts [58]. The global value chains have been reorganized as a result of the international trade-induced industrial shift, and the problem of GHG emission reduction must take into consideration the external pressures in the global dimension. Differences in countries’ ability to reduce GHG emissions, historical responsibility, and environmental perceptions, as well as the objective problem of “free-riding”, make a universally participatory and effectively-implemented multilateral climate agreement difficult to achieve yet, and the reality is that countries implement different levels of emission reduction policies or do not participate in emission reduction [59]. This asymmetric emission reduction policy may make GHG emissions an international investment, international trade as the medium of transnational transfer, in the economic level of each country, industrial differences in environmental policy influence, weakening the effect of emission reduction policies and affecting the effectiveness of global warming governance.

From the perspective of trade related to nuclear energy, scholars have also made some efforts. On the one hand, there is the impact of nuclear trade on national energy balances and environmental improvements. International nuclear trade is now of considerable importance for the energy balances of a number of countries. From the start, it has been regulated by bilateral or multilateral agreements, ones that always included conditions to obtain non-proliferation assurances with verification requirements. Nuclear trade indeed would have been impossible without the non-proliferation regime that has been developed [60]. As of 2018, nuclear plants are in operation or under construction in 30 countries around the world. Of these, only a few have industries to produce plants essentially from domestic resources, while the remaining are or have been importers. For example, France and Germany have been technology importers for the first pressurized-water reactors, but they are now self-sufficient and exporters. Japan has developed industrial self-sufficiency and could appear as an exporter, although it has not done so. The same will be the case in the future for more countries, including some developing ones, such as China and India, which are now almost self-sufficient in power plant production. These countries that are at the forefront of nuclear energy are also major drivers of global GHG reductions. Nuclear energy often represents a high-end clean energy technology, so promoting technological advances and transfers through global trade can better synchronize the goals of improving the ecological environment and ensuring sustainable development [61]. On the other hand, there are the national security risks and potential environmental problems associated with nuclear trade. As mentioned above, a nuclear leak is a serious national security incident [33]. The nuclear disasters at Chernobyl and Fukushima have been a wake-up call for the world [34,37]. At the same time, in the whole chain of nuclear power trade, the extraction of raw materials and the disposal of waste materials will bring unpredictable damage to the environment, which implies that nuclear power trade may indirectly have a negative impact on GHG management [21,41,42,43].

In general, existing studies have dialectically discussed whether nuclear power can curb GHG emissions. In these studies, the research areas are mostly concentrated in a single or in several countries, and the research methods are mostly life cycle assessment and case investigation. Meanwhile, the importance of international trade for GHG emission reduction was disclosed by many studies. However, few scholars have explored the spatial distribution of nuclear power product trade on a global scale, nor have they empirically analyzed the impact mechanism of nuclear power products on global GHG emissions from an empirical perspective. In this paper, we applied various methods such as social network, spatial analysis, and statistical modeling to fill this gap.

## 3. Materials and Methods

### 3.1. Data Source and Study Area

**Figure 1:**
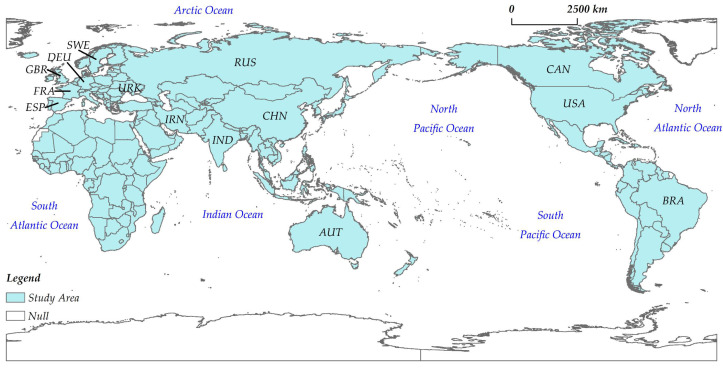
Study area: the spatial distribution of 191 countries involved in this paper.

The data of nuclear power product trade used in this paper are from the UN Comtrade database. UN Comtrade is a repository of official international trade statistics and relevant analytical tables. The study focused on extracting export trade flow data with 7187 (SITC Rev.3 Code), which includes nuclear reactors (71,871), non-irradiated fuel elements (71,877), and parts of nuclear reactors (71,878). The global GHG emission data are from the Climate Watch database established by the World Resources Institute (WRI). Climate Watch offers powerful insights and data on national climate plans, long-term strategies, and GHG emissions to help countries achieve their climate and sustainable development goals. Among the control variables, gross domestic product (GDP), foreign direct investment (FDI), urban population (UP), and national population (POP) are from the World Bank database, and the number of patent cooperation treaty (PCT) patents is from the World Intellectual Property Organization (WIPO) statistics database. All the above data were collected on a global scale from 2001 to 2018. Figure 1 shows the spatial distribution of 191 countries involved in this paper.

### 3.2. Construction of Trade Networks

Social network analysis, which originated from the sociological analysis of social relationships and the introduction of graph theory, is a well-established method for analyzing trade relationships among various actors and the structure of complicated trade systems [62]. A social network is a system of interactions, such as migration, collaboration, and trade, which constitutes a network [63]. Meanwhile, trade networks are social networks that developed as a result of commercial interactions. Social network analysis often explicitly describes the topology of an inter-country trade network, allowing for testing or relationship hypotheses, such as the spatial distribution of international trade linkages and the hierarchical structure of trading patterns across nations [64]. Thus, through the establishment and analysis of trade networks, the complex trade system can be seen as a whole to graphically and analytically represent trade pattern features and interactions among the countries in the world. The GNT network is constructed in the following way. 

The GNT network *N_t_* (*D_t_*, *E_t_*) is a weighted and directed network built to analyze the overall trade pattern and its evolution. The node set *D_t_* includes all the countries involved in the GNT from 2001 to 2018. Edge-set *E_t_* consists of the export trade flows among those countries (*D_t_*). Edge direction is indicated by the direction of trade (from exporter to importer). It should be noted that various indicators are used to represent the characteristics of trade network [65]. This study examines the node attribute, overall attribute, and hierarchical structure of the GNT network. It should be noted that the number of nodes and edges was counted in the *Gephi* software. 

#### 3.2.1. Network Node Attribute

Degree centrality (*N_d_*) does not consider the direction of trade. It is the sum of trade links that countries have in the network and represents a country’s position in the network [66].

(1) $$N_{d}\left(i\right)=\sum_{j=1}^{n}a_{ij}$$

where $a_{ij}$ denotes the matrix of countries’ connections. If country *i* has a trade link with country *j*, $a_{ij}$ = 1; otherwise, $a_{ij}$ = 0. 

Since the GNT network is a directed network, the degree centrality is divided into the in-degree and out-degree centrality. The in-degree centrality indicates the number of countries exporting nuclear power products to the country *i*, and the out-degree centrality indicates the number of countries exporting nuclear power products from the country *i*, so as to indicate the scale of cross-border nuclear power product trade in each country. In this paper, we counted only out-degree centrality based on the export trade data.

#### 3.2.2. Network Overall Attribute

Network density ($D_{g}$) is used to reflect the degree of closeness among the nodes in the network [67], which is the closeness of nuclear power product export trade links among countries, as identified in this study. $D_{g}$ measures the integrity and completeness of the network. The greater the density of the network, the closer the connection between the nodes in the network.

(2) $$D_{g}=x/[y(y-1)]$$

where *x* and *y* denote the total number of relationships and the total number of countries contained in the network, respectively.

Clustering coefficient (*C*) is used to measure the agglomeration degree of network connections. It is the average ratio of the number of direct connection edges of a node to the maximum number of possible connection edges.

(3) $$C\;=\frac{1}{n}\sum_{i=1}^{n}\frac{e_{i}}{k_{i}\left(k_{i}-1\right)}$$

where *n* denotes the total number of network nodes, $e_{i}$ represents the number of edges directly associated with node *i*, $k_{i}$ refers to the number of adjacency nodes, and $k_{i}\left(k_{i}-1\right)$ is the maximum number of possible edges.

Average path length (*L*) is a concept in network topology that is defined as the average number of steps along the shortest paths for all possible pairs of network nodes. It is a measure of the efficiency of information or mass transport on a network.

(4) $$L\;=\frac{\sum_{i\neq j}t_{ij}}{n(n-1)}$$

where $t_{ij}$ denotes the number of sides of the shortest path between country *i* and country *j*. By comparing the clustering coefficient and average path length of the global nuclear power product trade network with those of random networks, we can judge whether the global nuclear power product trade network has the small-world characteristic. 

#### 3.2.3. Network Hierarchical Structure Decision Model

The dominant flow analysis, proposed by Nystuen et al., considers that the strongest linkages of the nodes have more influence than other connections in the network [68]. It is mainly applied to the analysis of network hierarchy, such as world shipping [69], international student mobility [70], and global knowledge flow networks [71]. Nodes in the network are divided into three categories based on their size and degree of interaction, including dominant, subdominant, and affiliated. A dominant node is one whose maximum export flows to a node with a smaller scale than itself; a subdominant node sends the largest flow to dominant nodes and receives the strongest exports from a smaller node; an affiliated node is defined as a node without any of the largest flows from other nodes. In the GNT network, we used the dominant flow analysis to research the hierarchical structure.

### 3.3. Standard Deviation Elliptic Model

Standard deviational ellipse is an analytical method to characterize the spatial directional distribution of geographic elements, which can accurately reveal various characteristics of the spatial distribution pattern of geographic elements. It consists of four basic elements: coordinates of the center of gravity, rotation angle, standard deviation along the long axis, and standard deviation along the short axis, which represent the relative position of the spatial distribution pattern of geographic elements, the main trend direction of the development of geographic elements, and the degree of dispersion in the main and secondary directions, respectively [72].

### 3.4. Variables and Empirical Model

In this paper, GHG emissions were used as the dependent variable, and the trade volume of nuclear power products and network positions were used as the explanatory variable, respectively. Among them, referring to Gui et al. [73], we assigned different values to the network positions based on the results of the network hierarchical structure, where countries in the dominant position were assigned a value of 4, countries in the subdominant position were assigned a value of 3, countries in the affiliated position were assigned a value of 2, and other countries were assigned a value of 1. Taking into account the impact of other economic and environmental factors, we controlled several variables in the empirical models, such as the size of the economy (GDP, current prices) [74], external contact degree (FDI) [75], urban development level (UP) [74], population size (POP) [76], and technological development level (PCT) [77]. Table 1 lists and describes the dependent, explanatory, and control variables in the econometric models.

As GHG emissions are all non-negative integers and have discrete characteristics, the Poisson regression model can be used. However, the limitation of the model is that the dependent variables must be evenly dispersed (i.e., the mathematical expectations and variances are equal), whereas the data of GHG emissions are over-dispersed (i.e., the variance is greater than the mathematical expectation). Since this will lead to deviations in the results, the negative binomial regression model should be used [73,78]. Before we started, the Hausman test was performed for individual effects (i.e., country effects in this paper) to determine whether the fixed effect or random effect model should be used. The *p* value in the result was 0, which showed that the random effect model was strongly rejected. Therefore, we applied the fixed-effect negative binomial regression model in this paper. The regression model formula used to analyze the impact of GNT on GHG emissions is as follows:

(5) $${GHG}_{i,t}{=\alpha }_{i}{+\beta }_{1}{GNT}_{i,t}{+\beta }_{2}{GDP}_{i,t}{+\beta }_{3}{FDI}_{i,t}{+\beta }_{4}{UP}_{i,t}{+\beta }_{5}{POP}_{i,t}{+\beta }_{6}{PCT}_{i,t}{+\varepsilon }_{i,t}$$

where *i* denotes the country *i*, *t* represents the time series (2001–2018), *α* is a constant term, *β*_1_ … *β*_6_ are the coefficients to be estimated, and $\varepsilon_{i,t}$ is a random error term; the meanings of other variables are shown in Table 1. 

The regression model formula used to analyze the effect of network position on GHG emissions is as follows:

(6) $${GHG}_{i,t}{=\alpha }_{i}{+\beta }_{1}{POS}_{i,t}{+\beta }_{2}{GDP}_{i,t}{+\beta }_{3}{FDI}_{i,t}{+\beta }_{4}{UP}_{i,t}{+\beta }_{5}{POP}_{i,t}{+\beta }_{6}{PCT}_{i,t}{+\varepsilon }_{i,t}$$

where the meanings of the indexes are the same as Formula (5). 

We first performed a regression analysis for all the countries from a global perspective (the explanatory variable was the trade volume of nuclear power products, which addressed the second question of the paper). Then, based on the results of the hierarchical structure analysis, a regression analysis was performed with the network position of the countries as the explanatory variable, which was intended to address the third problem of this paper. 

## 4. Empirical Results and Analysis

### 4.1. Evidence from Spatio-Temporal Analysis

#### 4.1.1. Temporal Change 

**Figure 2:**
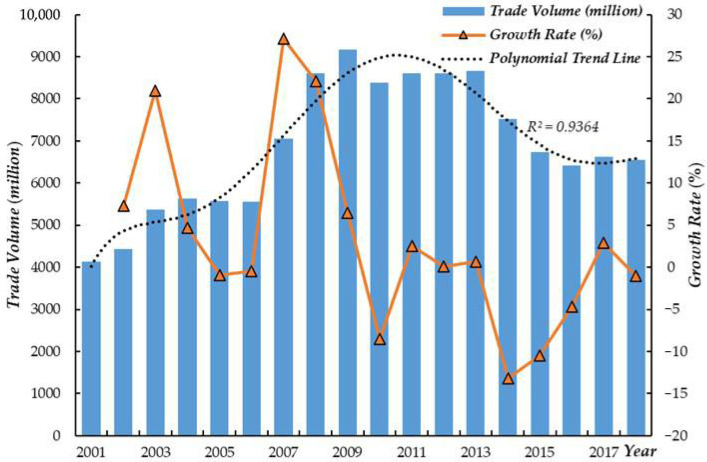
Changes in the GNT and growth rate, 2001–2018.

Figure 2 shows the growth of the volume of GNT from 2001 to 2018. On the whole, trade presents an increasing trend, with an average annual growth rate of 2.73%. The R^2^ of the polynomial trend line is 0.94, demonstrating a nonlinear fluctuation pattern in GNT growth. Specifically, it can be roughly divided into three stages: (1) Continuous rising period (2001–2009): During this period, unlike other commodity trade, the GNT was not affected by the 2008 financial crisis, and the trade volume increased steadily, from USD 4139.72 million in 2001 to USD 9167.65 million in 2009. (2) Gentle development period (2010–2013): In these four years, the volume of GNT had been maintained at about USD 8600 million. (3) Fluctuation declines period (2014–2018): Affected by the radiation water leakage of the Fukushima nuclear power plant in 2013, the volume of GNT began to decline in 2014, and this downward trend continued until 2018.

**Figure 3:**
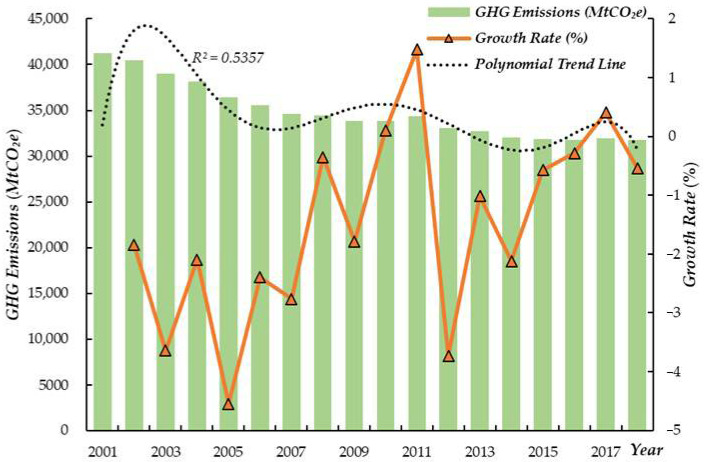
Changes in the GHG emissions and growth rate, 2001–2018.

Figure 3 shows the growth of the volume of GHG emissions from 2001 to 2018. Overall, the emissions showed a downward trend, with an average annual growth rate of −1.51%. The R^2^ of the polynomial trend line is 0.54, showing that GHG emissions fluctuated in a nonlinear manner. In terms of growth rate, the growth rate was negative between 2001 and 2009, which is inseparable from when the UNFCC came into effect in 1994. In 2010 and 2011, emission growth rates experienced a brief uptick before falling off a cliff again. From 2012 to the present, although the growth rate fluctuates and rises, emissions continue to develop in a decreasing trend generally. From the characteristics of the temporal change, it is clear that the trade volumes of nuclear power products rose and GHG emissions fell during the study period, which does not directly prove the mutual causality between the two, but provides support for the feasibility of this study.

#### 4.1.2. Spatial Evolution

**Figure 4:**
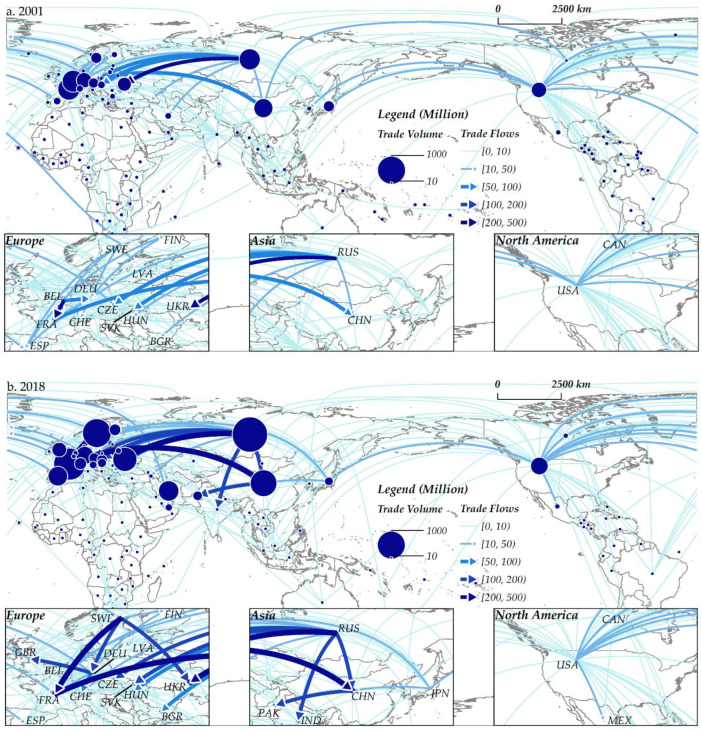
The spatial distribution of the GNT network from 2001 (**a**) to 2018 (**b**).

The GNT presented significant spatial heterogeneity, the gravity center shifted to the northeast slightly, and the largest trading country also changed from France to Russia (Figure 4). The top 10 exporters accounted for 98.9 to 99.2% of the total export volume. From 2001 to 2018, the main exporters were always developed countries in Europe, such as Russia, Sweden, Spain, Germany, and France. Apart from Europe, only a few countries (China and Japan in Asia, the US in North America) are major exporters. In the past 18 years, Russia’s trade capacity has increased rapidly, from USD 631.7 million in 2001 to USD 1144.2 million in 2018, with an average annual growth rate of 3.6%. At the same time, China’s imports and exports expanded further, from USD 469.3 million to USD 655.6 million, a growth rate of up to 39.7%. As a result, the gravity center of the spatial distribution of the GNT has shifted; the coordinates of the center point of the standard deviation ellipse moved from (47°38′1″ N, 29°48′23″ E) in 2001 to (49°15′47″ N, 35°43′48″ E) in 2018 in the northeast (Table 2). Overall, from 2001 to 2018, with the improvement of the countries’ abilities to export nuclear power products, a multipolar pattern with Europe, East Asia, and North America as the core has gradually taken shape. By analyzing the regional distribution of trading countries, it can be seen that these countries’ geographical positions show typical geopolitical characteristics. For example, Russia has close trade relations with Central and Eastern European countries formerly part of the Soviet Union, as does the US and North and South American countries. In addition to the countries mentioned above, Russia also exports nuclear power products to China, India, and Pakistan and cooperates with Iran, Turkey, and other countries with important geostrategic significance to invest in the construction of nuclear power plants [79].

The spatial structure evolution of the GNT network has both path-dependence and path-creation effect. Europe has always dominated the network, and Europe-Asia interconnections have always been the main axis of the network connection. The large-scale trade links in the network mostly occur within Europe, as well as between Russia, France, and Asian countries such as China, India, and Pakistan. Among the ten strongest trade links in 2001, five links were related to Russia and Western European countries (Figure 4). Belgium’s export volume to France ranks first in the world, reaching USD 469.3 million, followed by Russia→Ukraine (USD 225.5 million), France→China (USD 85.1 million), Russia→Slovakia (USD 74.7 million), Belgium→Germany (USD 60.4 million), Russia→Czechia (USD 54.3 million), France→Belgium (USD 50.6 million), Russia→Bulgaria (USD 41.9 million), and so on. In 2018, three global trade links remained firmly in the top ten, namely Russia→Ukraine (USD 343.3 million), France→China (USD 221.8 million), and Russia→Czechia (USD 119.0 million). This meant that the GNT was affected by the original trade state and had the characteristics of path-dependent development. In addition, the emergence of new trade pairs (including China, Pakistan, and India) in the top ten suggested that only a handful of countries in the world were able to achieve path-creation for nuclear power product trade.

**Figure 5:**
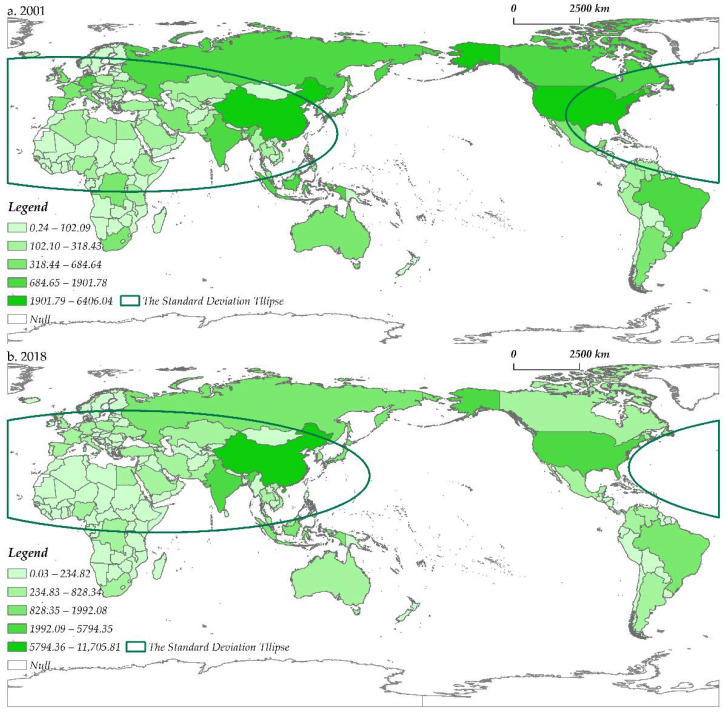
The spatial distribution of GHG emissions from 2001 (**a**) to 2018 (**b**).

The spatial distribution of global GHG emissions is shown in Figure 5. The ranking in the figure is based on the natural breaks’ method. The high value areas of global GHG emissions mainly show a spatial phenomenon such as the clustering of new industrialized countries or regions. In 2001, the top five countries in global GHG emissions were the US, China, Brazil, India, and Russia, which together emitted 15,970.7 Mt CO_2_e, or about 46.2% of all emissions. In addition, Japan, Germany, Canada, the United Kingdom, Italy, and other traditional economically-developed countries were also major “contributors” to GHG emissions. In 2018, most countries saw significant decreases in GHG emissions, such as the US from 6406.0 Mt CO_2_e in 2001 to 5794.4 Mt CO_2_e. However, emissions from developing countries with rapid economic growth have risen sharply, for example, from 4249.7 Mt CO_2_e to 11,705.8 Mt CO_2_e in China and from 1747.5 Mt CO_2_e to 3346.6 Mt CO_2_e in India. Such changes in spatial characteristics have driven a shift in the gravity center of the spatial distribution of global GHG emissions. As can be seen in the standard deviation ellipse analysis, the coordinates of the center point have moved remarkably from (29°36′8″ N, 14°38′36″ E) to (28°52′10″ N, 38°44′18″ E) to the southeast (Figure 5). In general, with the “explosive” economic and demographic growth of late-developing countries, the spatial pattern of emerging developing countries in Asia as the growth pole is basically “locked”.

Combining the spatial characterization of GNT and global GHG emissions, the two had some spatial reverse coupling. In terms of spatial distribution, the major trading countries of nuclear power products are concentrated in Europe, East Asia, and North America, while GHG emissions of Europe and North America as a whole declined significantly during the study period. This spatial contrast result implies that countries with high trade volumes in nuclear power products overlap in spatial distribution with countries with low GHG emissions, suggesting some spatial correlation between GNT and GHG emissions. At this point, the first research question of this paper is answered. In the next section, this paper provides a detailed analysis of this influence mechanism in statistical terms.

### 4.2. Evidence from Regression Analysis

#### 4.2.1. Results Estimating the Impact of GNT on GHG Emissions 

Before starting the regression, we performed analyses of the variables. Table 3 presents the descriptive statistics and a correlation matrix. In this paper, the average national GHG emission was 219.239 MtCO_2_e per year, with a standard deviation of 838.252 Mt CO_2_e, and the latter was more than three times the former, which reflects the heterogeneity of global warming. Equally, the standard deviations of explanatory and control variables were also greater than the average values. It shows that, in terms of economic size and technological and demographic development, the world is no longer “flat” [17,18], and global disparities are growing deeply [80,81,82]. The mean value of variance inflation factor is 1.59, and the maximum variance inflation factor value is 2.30, which indicates that multicollinearity is not significant in this paper.

Through the regression analysis, we verified whether the GNT plays an important role in the process of curbing GHG emissions. Model 1 in Table 4 was used as the basic model and shows the regression results of the five control variables. Model 2 presents the full model, which includes all control variables and explanatory variables. 

As shown in Model 1 of Table 4, it is not surprising that the coefficients of size of the economy, external contact degree, urban development level, and population size were all positive and statistically significant at the level of *p* < 0.01. Since the industrial revolution, the size of the economy has inevitably been accompanied by environmental “sacrifice”, a fact that has been corroborated by the findings of many scholars [83]. A high degree of external linkages increases national GHG emissions because, in the context of trade liberalization, higher levels of national external linkages encourage pollution-intensive industries to enter countries with lower environmental standards, thus turning them into so-called “pollution havens”, which will inevitably be accompanied by a surge in national GHG emissions [84]. Along with the rapid development of urbanization, the concentration of urban population has caused ecological imbalance, environmental degradation, and excessive GHG emissions. Similarly, neo-Malthusianism argues that the growing population will lead to increasing environmental pollution [85]. Technological development level is also significantly correlated with GHG emissions and has a negative coefficient. High-tech countries tend to have a higher awareness of environmental protection and environmental standards, so this variable shows a significant negative correlation. 

The second central question of this paper was whether trade in nuclear power products has a significant impact on GHG emissions on a global scale. As shown in Model 2 in Table 4, the coefficient of the GNT was negative and statistically significant at *p* < 0.1, which means that countries with developed trade in nuclear power products have lower GHG emissions. With all other variables held constant in Model 2, a 1% increase in nuclear power product trade was associated with a 0.677% decrease in GHG emissions. Nuclear power products are inherently technology-intensive, so the large nuclear power trading nations are usually accompanied by top technology levels. It is interesting to note that the coefficient for GNT was the highest of all variables, indicating that nuclear power product trade had the greatest impact on GHG emissions. This result is also in line with the first branch in the literature review. As noted by Kharecha et al., global nuclear power has prevented an average of 1.84 million air pollution-related deaths and 64 gigatons of CO_2_-equivalent (Gt CO_2_-eq) GHG emissions that would have resulted from fossil fuel burning [86]. Hence, the second research question of this paper is verified and explained.

#### 4.2.2. Results Estimating the Impact of GNT Networks on GHG Emissions

In this section, we use network position as the explanatory variable to explore the mechanism of GNT network influence on GHG emissions. 

First, we need to understand the basic characteristics of GNT network structure development. As shown in Table 5, while the node number increased from 108 in 2001 to 111 in 2018, the total number of links reduced from 314 in 2001 to 307 in 2018, indicating that nuclear power product trade between countries is sparser. The average number of countries connected to each country (degree centrality) decreased slightly (from 5.815 to 5.532), as did network density (from 0.027 to 0.025), which further confirmed the thinning trend of the trade network. At the same time, the out-degree centrality also decreased from 2.907 to 2.766, indicating that the overall exit link tightness of the GNT network decreased. The decline of the clustering coefficient (from 0.261 to 0.202) shows that trade resources are no longer concentrated in several countries. We obtained the theoretical values of clustering coefficient (0.021) and average path length (2.487) of a random network constructed on the basis of the GNT network in 2018. However, the actual network has a higher clustering coefficient (0.202) and average path length (2.854), denoting that the small-world characteristic is not significant in the GNT network.

Second, we identified the network positions of countries in the GNT network by analyzing the hierarchical structure and assigning values to the countries in different positions. Specifically, we assigned a value of 4 to the country in the dominant position, a value of 3 to the country in the subdominant position, a value of 2 to the country in the affiliated position, and a value of 1 to the other countries. 

**Figure 6:**
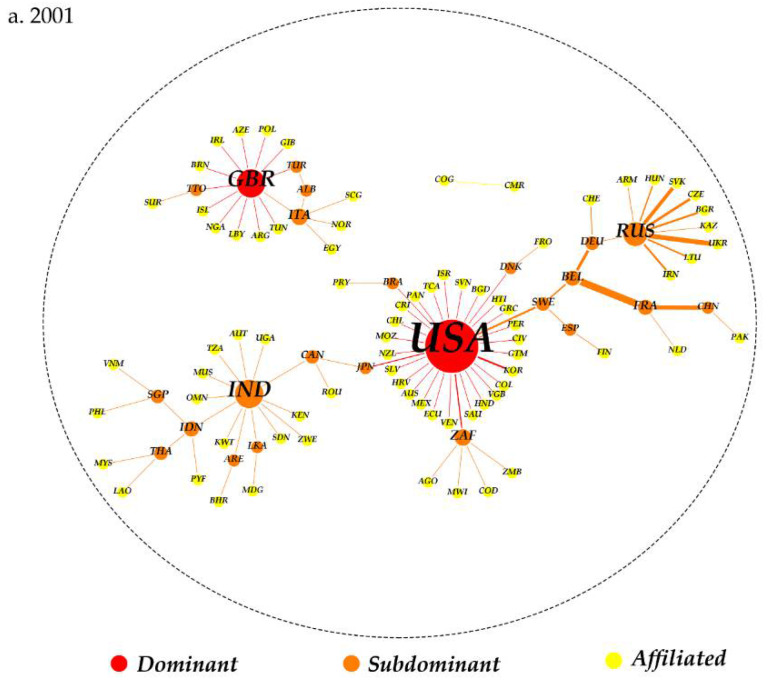

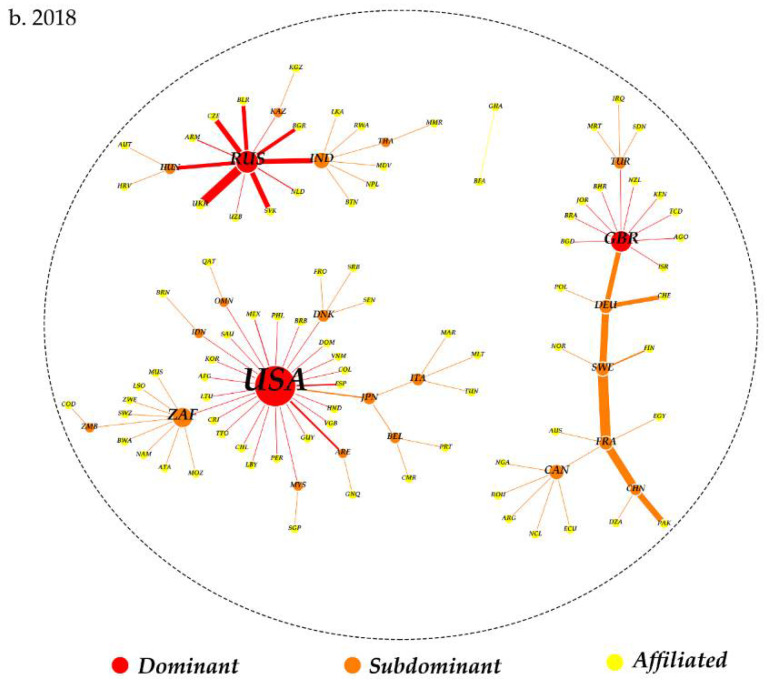
The hierarchical structure of the GNT network in 2001 (**a**) and 2018 (**b**). (Note: The size of the nodes changes with the degree centrality; the thickness of the edge varies with nuclear power product exports; and the color of the line is consistent with that of the node—that is, this link belongs to a higher-level node).

Figure 6 shows the evolution of the hierarchical structure in the GNT network from 2001 to 2018. In 2001, except for two isolated nodes (Cameroon and Congo), the entire trade network was divided into two different sub-networks, including a fully connected network of 78 countries and a local small network. The largest sub-network led by the US consisted of countries all over Europe, Asia, Africa, and the Americas. Eighteen countries, including Russia, India, France, Germany, and China, were the subdominant nodes in the largest sub-network. The second sub-network, dominated by the UK, included eighteen countries geographically concentrated in Europe and Asia, among which the representative countries were Italy, Albania, and Turkey, which may be influenced by the geographical proximity effect [87]. In 2018, significant changes occurred in the dominant economies and the overall structure. The largest sub-network has undergone fission, and its space coverage has shrunk, but the position of the US has been strengthened. The largest sub-network still had the deepest spatial breadth, and the number of members in the network dropped to fifty-one countries. Many countries are directly influenced by the US and actively maintain and expand trade cooperation with the American market. Some countries, with Russia as the core, have separated from the largest sub-network and formed an independent sub-network. With the rapid development of nuclear technology, Russia has become the country with the largest dominant flows and is the primary trading partner of many countries. This is a typical regional trade network with significant geopolitical meaning. The sub-network with the links between Britain, France, Germany, and Sweden as the backbones has also attracted a large number of countries to join. The number of countries in sub-dominant clusters rose to six in this sub-network. The geographical dispersion of these subdominant nodes indicated that regional trade hubs were emerging in the GNT network. 

From the evolution of the GNT network structure, there is an obvious uneven sharing of resources and hierarchical differences in the global promotion of nuclear power products. The application of nuclear power products in the dominant countries in the GNT network is bound to be better than that in the affiliated countries, which is bound to promote the spatial phenomenon of differentiation in the governance of global GHG emissions. Therefore, to analyze the impact of GNT on global GHG emissions, we need not only an overall analysis from a global perspective, but also a differentiated study of countries in different network positions.

Third, we introduced a variable, POS, to measure the network position of the country. The regression results are shown in Table 6. We did not perform regression analysis on the control variables again because this is a duplication of the previous section. 

In Table 6, the correlations of all control variables remained consistent with those in Table 4, but the coefficients of those, except for population size, decreased. The *β* coefficient of POS was −0.044 and statistically significant at *p* < 0.01, which indicates that the higher the value of network position in the GNT network, the lower the GHG emissions (i.e., the network positions have the suppressive effect on GHG emissions). With all other variables held constant in Table 6, a 1% increase in network positions reduced GHG emissions by 0.044%. This corresponds with the findings of Jacoby and Paltsev who, based on a full study of alternative scenarios of nuclear power exit that consider the influence of potential policies to limit GHG emissions, stated that a US nuclear power exit would increase GHG emissions sharply [88]. Therefore, this result answered, to some extent, the third research question of this paper, which is that countries with dominant positions emit less GHGs compared to affiliated countries. 

#### 4.2.3. Robustness Test

In order to verify the validity of the results in Table 4 and Table 6, we further performed a robustness test in this section. Before conducting the robustness test, a clarification was necessary. In the previous analysis, we used POS to denote the network position. In this part, we divided the countries into dominant positions (including the previously dominant nodes and the sub-dominant nodes) and affiliated positions according to the results in the hierarchical structure analysis and conducted regression analyses for each. This operation not only allows robustness testing of the overall analysis results (Table 4), but also validates the validity of the network position and makes the heterogeneity tests for regression results.

First, in Model 1 and 2 of Table 7, the correlations of all variables remained the same as in the models of Table 4. As can be seen, the coefficient for GDP was much higher than that in Table 4, indicating that size of the economy is more sensitive to GHG emissions in the dominant countries, which are mainly composed of developed countries. POP did not pass the significance test, but its regression coefficient has improved. In model 4, we added the explanatory variables, and the results are shown in Table 6. The *β* coefficient of GNT was −0.728, proving that trade in nuclear power products has a greater dampening effect on GHG emissions in the countries with dominant positions. With all other variables held constant in model 4, a 1% increase in nuclear power product trade was associated with a 0.728% decrease in GHG emissions. This result is in line with comparable prior studies that the GHG emissions from the use of nuclear power products are close to zero, and it can be said that nuclear power products do not directly increase GHG emissions [1,30,31,32]. It can be seen that the influence of GNT on GHG emissions is greater in Table 7 than in Table 4. However, this is only relative to the overall regression results for all countries worldwide, and we subsequently transferred the research object to countries with affiliated positions to be able to obtain more scientific results.

Second, we performed regression analysis for countries with affiliated positions in Models 3 and 4 of Table 7. While the correlation of all variables remained consistent with all previous models, something interesting happened. Provided that they were statistically significant at the level of *p* < 0.01, the *β* coefficients for POP and PCT were the highest, indicating that population size and technological development level have the greatest impact on GHG emissions in the countries with affiliated positions, which mainly consisted of developing economies. Actually, developed countries have been the primary source of carbon dioxide in the past, but rapid population and economic growth in the developing world is expected to raise its emission rate above that of the industrialized countries during most of the twenty-first century. As Shi points out, the increased population in less developed countries will contribute twice as much to emissions as the increased population in developed countries, because they are emulating a higher standard of living and more energy use [89]. In terms of the negative correlation effect of PCT, for developing countries that are technologically backward, an increase in the level of technological development can be more effective in delivering environmental benefits. The *β* coefficient of GNT was −0.342, which had the highest statistical significance (*p* < 0.05) compared to the previous models. With all other variables held constant in Model 6, a 1% increase in nuclear power product trade was associated with a 0.342% decrease in GHG emissions. At the same time, the effect of GNT on GHG emissions was weaker in the affiliated countries compared to the dominant countries. Therefore, our previous findings are supported by the robustness test.

In conclusion, through our detailed regression analysis, the core research questions of this paper have been answered. First, nuclear power product trade has a significant negative effect on global GHG emissions. Compared with other control variables, the suppressive effect of nuclear power product trade was the strongest. Second, network positions have a significant impact on GHG emissions, and countries with dominant positions are better able to manage GHG emissions through nuclear power product trade. All the above regression analyses passed the robustness test. At the same time, we found in the robustness test that the impact effect was different among different countries, indicating that the impact of nuclear power product trade on GHG emissions is also characterized by heterogeneity in the statistical analysis.

## 5. Discussion

Nuclear power product, a controversial product, has long been a focus of scholarly discussion. First, this paper described the spatial and temporal evolution of the GNT and explored the spatial reverse coupling of GNT and global GHG emissions. Second, we empirically analyzed the impact of GNT on GHG emissions from both an overall and network perspective. This study is different from those of other scholars, such as Sim et al. and Ren et al., who studied the impact of nuclear power on GHG emissions based on life cycle analyses in several countries [31,32]. In their study, they confined themselves to individual cases, and although detailed calculations of GHG emissions from nuclear power in applications were made, this hardly provided an effective guide to the governance of global GHG emissions. In contrast, in this study, we analyzed the global trade pattern of nuclear power products among 191 countries and explored the factors influencing GHG emissions on a sub-scale basis, which not only portrays the spatial distribution of global environmental protection factors, but also can provide effective suggestions for global environmental governance. While some scholars have analyzed the international trade patterns of nuclear power products and others have explored the factors influencing GHG emissions [90,91,92], research combining the two is lacking, especially since nuclear power products have always been an effective means of reducing GHG [38,39,40]. Of course, our study also has certain shortcomings, as the consideration of control variables may need to be improved. For example, policy factor variables are missing from our variables, due to the limited availability of data. 

An analysis of the impact of global trade on GHG emissions also brings new thinking to the table. Globalization has led to an increasing geospatial separation of production and consumption, and international trade has led to the transfer of GHG along with trade flows. In the context of global carbon governance and global climate policy, the international capitalist system has imposed unequal responsibility for international GHG governance on some underdeveloped countries, resulting in huge pressure on GHG emission reduction of emerging economies that receive international industrial transfers. The emerging economies that receive international industrial transfer are under enormous pressure to reduce GHG emissions. Simultaneously, the inequality of carbon emission recognition standards and carbon emission responsibility division in international carbon governance actually exists, and emerging economies are facing the double pressure of resources, environment, and economic development in taking over industrial transfer through international trade; thus, developing countries need to take an active role in international carbon emission responsibility recognition. Developing countries should study new emission reduction policies, accept technology transfer and international assistance from developed countries, and develop renewable and clean energy industries (i.e., nuclear power industries). Developed countries should raise their awareness of responsibility, actively assume responsibility for global GHG governance, strengthen technology transfer and international assistance, and use global diversified emission reduction strategies to continuously achieve emission reduction targets.

The existence of an environmental Kuznets curve (EKC) hypothesis regarding the impact of nuclear power trade on greenhouse gas emissions has been widely discussed. In a study for China, Dong et al. [93] considered the effects nuclear power on environmental pollution, taking fossil fuels and renewable energy into account across 1993–2016. Results indicated the existence of an EKC hypothesis and concluded that nuclear energy positively contributes to pollution mitigation. Baek and Kim [94] considered the important of nuclear energy within the framework for Korea for the data between 1971–2007 and 1978–2007. The study suggested that nuclear energy reduces environmental pollution, with the confirmation of the EKC hypothesis in both the short and long runs. Moreover, Sarkodie and Adams [8] studied the effects of nuclear energy, renewable energy, and CO2 emissions for data spanning between 1971 and 2017 in South Africa. The results revealed that nuclear energy escalated pollution with the support of the EKC hypothesis. There are other studies that hold a different view. Another study [95] projected the EKC hypothesis, controlling the role of electricity generation from nuclear energy for the data spanning 1960–2003 in France and concluded that nuclear energy contributes to pollution mitigation and invalidated the EKC hypothesis. Contrarily, Mbarek et al. [96] found that nuclear energy does not play any significant role in carbon emission reduction and could not find proof for the EKC hypothesis in a panel of developing and developed countries. Similarly, Saidi and Ben Mbarek [5] found the same results for evidence for nine developed countries. Likewise, Jin and Kim [97] acquired similar results for 30 countries between 1990 and 2014. Since the focus of this paper was on the combination of spatial, network, and empirical methods from a global scale, the EKC has not yet been tested, but this does point the way to future research.

## 6. Conclusions

Using social network analysis, spatial analysis, correlation analysis, and fixed effect negative binomial regression in this paper, the structure of nuclear power product trade and its effect on GHG emissions in 191 countries were studied, and the main findings are as follows: 

First, the global trade volume of nuclear power products showed a non-linear fluctuating growth. After the Fukushima nuclear power plant accident in Japan, the GNT was dealt a serious blow, but in recent years, as the international situation has changed, nuclear power may once again become a new energy generation method strongly promoted by the government. For instance, the European Parliament adopted a new green energy investment program that includes nuclear power in the green power category in February 2022, making qualifying investments in nuclear power “green investments”. At the same time, the French government announced its plan to redevelop nuclear power by building six new European pressurized water nuclear reactors and increasing the service life of all existing nuclear reactors to more than 50 years on a safety basis. This means that there will be a new period of upward development in the global trade of nuclear power products in the future. Meanwhile, global GHG emissions are gradually decreasing, indicating that GHG emission reduction and strengthening global warming governance have become a global consensus.

Second, along with the rapid rise of several newly industrialized economies, the world geography of nuclear power product trade is undergoing a spatial reconfiguration phenomenon. The rapid narrowing of the trade gap between China and developed countries is driving the spatial distribution of GNT to shift slightly eastward. Overall, the multipolar pattern of “Europe-East Asia-North America” is basically “locked”. The evolution of the spatial structure of the GNT network has the dual effects of path-dependence and path-creation. In addition, the geographical distribution of countries with close trade links in nuclear power products has typical geopolitical characteristics [61]. The high value areas of global GHG emissions are also mainly manifested by spatial phenomena such as the clustering of newly industrialized countries or regions. There is a certain spatial reverse coupling characteristic between GNT and GHG emissions. This result answered, exactly, the first question raised at the beginning of this paper.

Third, based on our statistical analysis, we found that GNT has a significant negative effect on global GHG emissions. Of all the variables considered, nuclear power product trade ha the largest effect on GHG emissions, far outweighing the factors representing the level of economic and population size. Immediately after, we analyzed the GNT network position by applying the hierarchical structure decision model. In the hierarchical structure, newly dominated nodes were rapidly developing, and the polarization of networks became increasingly remarkable. The US, Russia, and the UK were the largest dominant nodes in each of the three sub-networks, indicating that regional trade centers are emerging in the GNT network and that network resource allocation is heavily concentrated in a few hubs. The regression results show that the coefficients of the network position in the model were negative and statistically significant in panel data estimates. The importance of network position has been revealed by many scholars [73,98,99], so we verified whether network position affects GHG emissions in the GNT network. The results showed that nuclear power product trade is better able to curb GHG emissions in countries with the dominate positions compared to those with affiliated positions, which is a reflection of heterogeneity. These results answered the second and third questions of this paper. All regression results were tested for robustness.

This paper examined the impact of nuclear power products on global GHG emissions from the perspective of global trade. The import and export trade volumes of nuclear power products of a country usually represent the participation of this country in the international division of labor system and the degree of development of its nuclear power industry. Generally speaking, the higher the country’s trade volume of a certain product, the more advanced the country’s industry development and the more widely used the product is. This characteristic is particularly present in industries with high trade and technological barriers (e.g., nuclear power industry). Therefore, in this paper, we argued that the application of nuclear power products is more proficient in countries that have high trade volumes of nuclear power products. For example, in 2018, France ranked second in the world in terms of nuclear power products trade, and more than 70% of its domestic electricity supply was derived from nuclear power [21,23]. In terms of the global distribution of GHG emissions, most of these countries are in the low value area. Although this phenomenon may be the result of multiple influences (e.g., countries with high trade in nuclear power products are technologically advanced and the population is environmentally conscious), the results at least suggest some potential linkage mechanism between GNT and GHG. After controlling for relevant variables, our empirical analysis found that trade in nuclear power products had the largest effect on GHG emissions, in line with our previous hypothesis. 

This paper provides important implications for policymakers. First, trade in nuclear power products has been sluggish in recent years [34]. The current development of nuclear power is still very inadequate compared to its role, and the potential needs to be tapped. Therefore, the GNT should be encouraged, supported, and facilitated by the governments. Second, trade in nuclear power products has significant spatial heterogeneity. The governments of the dominant countries in the GNT network should take responsibility for promoting the development of nuclear power products, adjusting their nuclear power development plans, and actively cooperating in trade [26]. Under the premise of ensuring safety, the life span of nuclear power products should be extended as much as possible to maximize the environmental benefits brought by nuclear power product trade. Third, national dominance in the GNT network facilitates the effect of trade in nuclear power products on GHG emissions. Among the 191 countries studied in this paper, only 107 countries participate in the GNT, so the dominant countries should expand the trade direction as much as possible and bring more countries to join in. On the one hand, it can improve the vitality of the global economy, and on the other hand, it can improve the global warming governance as a whole.

## Figures and Tables

**Table 1:** Variable descriptions and data sources.

Variable	Description	Source
Dependent variable		
GHG	National GHG emissions	Climate Watch
Explanatory variable		
GNT	The trade volume of nuclear power products in a country	UN Comtrade
POS	Dominant: 4; subdominant: 3; affiliated: 2; other: 1	
Control variables		
GDP	Gross national product of a country (USD, billions)	Word Bank
FDI	Foreign direct investment within a country (USD, millions)	Word Bank
UP	Average urban population of a country (thousands)	Word Bank
POP	Total population of a country (millions)	Word Bank
PCT	The number of PCT patents in a country (thousands)	WIPO

**Table 2:** Gravity center of spatial distribution for the GNT from 2001 to 2018.

Parameters	2001	2018
Center X	47°38′1″ N	49°15′47″ N
Center Y	29°48′23″ E	35°43′48″ E
X standard distance	84.344	71.935
Y standard distance	15.413	15.282
Rotation	88.911	88.847

**Table 3:** The descriptive statistics and the correlation matrix of all variables.

Variables	Mean	*SD*	1	2	3	4	5	6
GHG	219.239	838.252						
GNT	35.967	162.080	0.337 ***					
GDP	322.281	1356.077	0.789 ***	0.398 ***				
FDI	896.004	3649.205	0.596 ***	0.290 ***	0.686 ***			
UP	18.381	60.488	0.407 ***	0.271 ***	0.358 ***	0.283 ***		
POP	35.837	134.890	0.802 ***	0.203 ***	0.452 ***	0.363 ***	0.409 ***	
PCT	72.108	482.696	0.673 ***	0.290 ***	0.911 ***	0.550 ***	0.256 ***	0.332 ***

*** *p* < 0.01.

**Table 4:** Analysis results of the influence mechanisms of GNT on global GHG emissions.

Variables	Model 1		Model 2	
	Coefficient	*SD*	Coefficient	*SD*
GDP	0.117 ***	0.026	0.118 ***	0.026
FDI	0.051 ***	0.009	0.051 ***	0.009
UP	0.038 ***	0.002	0.038 ***	0.002
POP	0.175 ***	0.021	0.180 ***	0.022
PCT	−0.096 ***	0.011	−0.090 ***	0.012
GNT			−0.677 *	0.362
Constant	1.465 ***	0.209	1.429	0.211
Observations	3438		3438	
Number of countries	191		191	
Log likelihood	−11,284.92		−11,283.11	
Wald *χ*^2^	1472.71		1472.07	

*** *p* < 0.01, * *p* < 0.1.

**Table 5:** The characteristics of GNT networks in 2001 and 2018.

	2001	2018
Node number	108	111
Edge number	314	307
Degree centrality	5.815	5.532
Out-degree centrality	2.907	2.766
Network density	0.027	0.025
Clustering coefficient	0.261	0.202
Average path length	2.673	2.854

**Table 6:** Results of the models of the impact of network position on GHG emissions.

Variables	Coefficient	*SD*
GDP	0.041 ***	0.012
FDI	0.011 ***	0.003
UP	0.003 ***	0.001
POP	0.253 ***	0.067
PCT	−0.041	0.038
POS	−0.044 **	0.205
Constant	0.845	0.604
Observations	3438	
Number of countries	191	
Log likelihood	−647.62	
Wald *χ*^2^	269.48	

*** *p* < 0.01, ** *p* < 0.05.

**Table 7:** Analysis results of the influence mechanisms of GNT on global GHG emissions in different positions.

Variables	Dominant Positions		Affiliated Positions	
	Model 1	Model 2	Model 3	Model 4
	Coefficient (*SD*)	Coefficient (*SD*)	Coefficient (*SD*)	Coefficient (*SD*)
GDP	0.929 *** (0.191)	0.990 *** (0.194)	0.097 *** (0.025)	0.099 *** (0.025)
FDI	0.032 ** (0.016)	0.033 ** (0.016)	0.054 *** (0.010)	0.053 *** (0.010)
UP	0.027 *** (0.004)	0.026 *** (0.004)	0.030 *** (0.008)	0.029 *** (0.007)
POP	0.408 (0.407)	0.415 (0.408)	0.184 *** (0.032)	0.190 *** (0.033)
PCT	−0.062 *** (0.018)	−0.054 ** (0.018)	−0.236 *** (0.064)	−0.265 *** (0.063)
GNT		−0.728 * (0.415)		−0.342 ** (0.129)
Constant	0.730 *** (0.500)	0.580 *** (0.507)	1.477 *** (0.293)	1.435 *** (0.299)
Observations	522		3006	
Number of countries	29		167	
Log likelihood	−2543.08	−2541.49	−9099.62	−9095.78
Wald *χ*^2^	539.01	530.07	203.64	213.81

*** *p* < 0.01, ** *p* < 0.05, * *p* < 0.1.

## Data Availability

UN Comtrade database, Climate Watch database, World Bank database, World Intellectual Property Organization statistics database.
